# Genome-Wide Association Analysis Reveals Loci and Candidate Genes Involved in Fiber Quality Traits Under Multiple Field Environments in Cotton (*Gossypium hirsutum*)

**DOI:** 10.3389/fpls.2021.695503

**Published:** 2021-08-05

**Authors:** Xiaohui Song, Guozhong Zhu, Sen Hou, Yamei Ren, Muhammad Waqas Amjid, Weixi Li, Wangzhen Guo

**Affiliations:** State Key Laboratory of Crop Genetics and Germplasm Enhancement, Cotton Germplasm Enhancement and Application Engineering Research Center (Ministry of Education), Nanjing Agricultural University, Nanjing, China

**Keywords:** fiber quality traits, genome-wide association study, upland cotton, single nucleotide polymorphism, quantitative trait loci

## Abstract

Fiber length, fiber strength, and fiber micronaire are the main fiber quality parameters in cotton. Thus, mining the elite and stable loci/alleles related to fiber quality traits and elucidating the relationship between the two may accelerate genetic improvement of fiber quality in cotton. Here, genome-wide association analysis (GWAS) was performed for fiber quality parameters based on phenotypic data, and 56,010 high-quality single nucleotide polymorphisms (SNPs) using 242 upland cotton accessions under 12 field environments were obtained. Phenotypic analysis exhibited that fiber length (FL) had a positive correlation with fiber strength (FS) and had a negative correlation with fiber micronaire (Mic). Genetic analysis also indicated that FL, FS, and Mic had high heritability of more than 80%. A total of 67 stable quantitative trait loci (QTLs) were identified through GWAS analysis, including 31 for FL, 21 for FS, and 22 for Mic. Of them, three pairs homologous QTLs were detected between A and D subgenomes, and seven co-located QTLs with two fiber quality parameters were found. Compared with the reported QTLs, 34 co-located with previous studies, and 33 were newly revealed. Integrated with transcriptome analysis, we selected 256, 244, and 149 candidate genes for FL, FS, and Mic, respectively. Gene Ontology (GO) analysis showed that most of the genes located in QTLs interval of the three fiber quality traits were involved in sugar biosynthesis, sugar metabolism, microtubule, and cytoskeleton organization, which played crucial roles in fiber development. Through correlation analysis between haplotypes and phenotypes, three genes (*GH_A05G1494, GH_D11G3097*, and *GH_A05G1082*) predominately expressed in fiber development stages were indicated to be potentially responsible for FL, FS, and Mic, respectively. The *GH_A05G1494* encoded a protein containing SGS-domain, which is related to tubulin-binding and ubiquitin-protein ligase binding. The *GH_D11G3097* encoded 20S proteasome beta subunit G1, and was involved in the ubiquitin-dependent protein catabolic process. The *GH_A05G1082* encoded RAN binding protein 1 with a molecular function of GTPase activator activity. These results provide new insights and candidate loci/genes for the improvement of fiber quality in cotton.

## Introduction

Cotton is cultivated in many countries globally because it provides the most important natural textile fiber (Chen et al., 2007). Cotton has four cultivated species: namely, the two diploid species *Gossypium herbaceum* and *Gossypium arboretum*, and the two tetraploid species (*Gossypium hirsutum* and *Gossypium barbadense*) (Huang et al., 2017). The upland cotton (*Gossypium hirsutum*), in particular, contributes to more than 95% of total cotton production (Su et al., 2019). Cotton fiber has been used in various textile specifications, with the benefits of warmth, moisture absorption, breathability, and wearing comfort. However, with the advancement of textile technology, low-quality cotton fiber has difficulty meeting the machining requirements; therefore, improving fiber quality has become an essential goal for modern cotton breeding.

The fiber quality traits, namely, fiber length (FL), fiber strength (FS), and fiber micronaire (Mic), are complex quantitative traits regulated by multiple genes (Paterson et al., 2003; Shen et al., 2007). Several quantitative trait loci (QTLs) for fiber quality traits have been identified by using bi-parental linkage mapping analysis (Said et al., 2013, 2015), which provided the candidate loci for the improvement of fiber quality traits. However, due to the low density of molecular markers, these QTLs often flanked large genetic regions and made mining key genes challenging. Based on the high-density single nucleotide polymorphisms (SNPs), genome-wide association study (GWAS) has been identified as an effective tool for discovering QTLs and genes associated with target traits in various crops such as wheat (Juliana et al., 2019), rice (Chen et al., 2014), soybean (Lu et al., 2020), and maize (Wang et al., 2020).

Several studies have been reported in cotton. A total of 46 significant SNPs and 612 unique candidate genes associated with fiber quality traits were identified through GWAS by using 719 diverse upland cotton accession in multiple environments (Sun et al., 2017). Su et al. (2020) also reported 25 stable QTLs related to five fiber quality traits [FL, FS, Mic, fiber uniformity (FU), and fiber elongation (FE), respectively] under normal and salt environments using 279 sea island cotton accessions through GWAS. Ma et al. (2018), on the other hand, found 7,383 SNPs involved significantly in fiber quality using 419 genotypes of upland cotton in 12 environments. Thus, the QTLs and candidate genes identified through GWAS exhibit significant potential in cotton breeding for fiber quality improvement.

In this study, a total of 242 upland cotton accessions grown in 12 different natural environments were used for GWAS to identify QTLs/genes for three fiber quality parameters: FL, FS, and Mic. A total of 67 stable QTLs were identified, and key genes contributing to fiber quality were analyzed. These stable QTLs/genes can be used to develop cotton varieties with higher fiber quality.

## Materials and Methods

### Plant Materials

In this study, a total of 242 upland cotton accessions were used for GWAS. These accessions were collected from different regions of China and introduced from the United States. All accessions were preserved and planted by the Cotton Research Institute of Nanjing Agricultural University. All necessary permits for planting and investigating natural populations were obtained from Nanjing Agricultural University, China.

With random block design and two replicates for each accession in every environment, all the accessions were planted in 12 field environments from 2011 to 2018. The 12 environments were Xinjiang-Korla, Henan-Xinxiang, and Henan-Nanyang in 2011, 2012, and 2013, respectively, Xinjiang-Shawan in 2017, and Jiangsu-Yancheng and Anhui-Dangtu in 2018 (Supplementary Table 1). All agronomic practices were applied following the local management. In each environment, 10 plants for each accession were selected randomly at 70% boll opening, and 20 well-developed bolls with 2 bolls/plant from the middle branches were collected to measure FL (mm), FS (cN·tex^−1^), and Mic. The three fiber quality parameters were measured by the Center of Cotton Fiber Quality Inspection and Testing, Ministry of Agriculture and Rural Affairs.

### Statistical Analysis

The Best Linear Unbiased Prediction method using the “lmer” function in the lme4 package was used to estimate the breeding value of each accession (Bates et al., 2014). The broad-sense heritability (*h*^2^) of each trait was calculated using SAS software (Institute, 2013). Statistical analyses of the phenotypic data were conducted using SPSS 25.0 software. The correlation coefficient and its significance among traits based on the Best Linear Unbiased Prediction (BLUP) value were calculated using the R software “PerformanceAnalytics” package.

### SNP Genotyping

Young leaves were collected from 242 accessions for DNA extraction (Paterson et al., 1993), and CottonSNP80K array containing 77,774 SNPs was used for genotyping (Cai et al., 2017). Under the Illumina protocols, qualified DNA was hybridized to the CottonSNP80K array. The array was then scanned by the Illumina iScan array scanner, and the GenomeStudio Genotyping software (V2011.1, Illumina, Inc.) was used to cluster SNP alleles and genotyping. To determine the physical location of SNP on the reference genome, we mapped the probe sequences back to the TM-1 V2.1 genome (Hu et al., 2019). In total, 56,010 high-quality SNPs [calling rate ≥0.9 and minor allele frequency (MAF) ≥0.05] were obtained for subsequent analysis.

### GWAS Analysis

GWAS was conducted under the framework of multi-locus random-SNP-effect mixed linear model (mrMLM). The mrMLM was used in the “mrMLM” package of R software, including six association methods: mrMLM (Wang et al., 2016), FASTmrMLM (fast multi-locus random-SNP-effect mixed linear model) (Tamba and Zhang, 2018), FASTmrEMMA (fast multi-locus random-SNP-effect efficient mixed model association) (Wen et al., 2017), pLARmEB (polygenic-background-control-based least angle regression plus empirical Bayes) (Zhang et al., 2017), pKWmEB (polygenic-background-control-based Kruskal-Wallis test with empirical Bayes) (Ren et al., 2018), and ISIS EM-BLASSO (iterative modified-sure independence screening Expectation-Maximization-Bayesian least absolute shrinkage and selection operator) (Tamba et al., 2017). For the critical LOD (log of odds) scores of all methods, all the QTNs with their LOD scores ≥3.0 were regarded as significant QTNs. The Q+K model was also adopted for GWAS analysis. According to our previous study (Zhu et al., 2019), the K value is 3, and the population structure was calculated using admixture 1.3.0 software. The population relationship K matrix was calculated using the “mrMLM” package.

### Identification of QTLs and Candidate Gene

Due to the uneven distribution of the genotyped SNPs, we selected the lowest LD (linkage disequilibrium) of the chromosome, ~200 kb, on both sides of the physical position of quantitative trait nucleotides (QTN) as the initial QTL (Su et al., 2020). The overlapping QTL was then merged the as the same QTL. When the merged QTL contained or overlapped with the previously reported QTL identified with marker location, we regarded them as the co-located QTL. According to the physical location of the QTL, candidate genes were searched from the reference genome (Hu et al., 2019). To further determine the relationship between genes and fiber quality traits, we downloaded the transcriptome profiles of TM-1 tissues from National Center of Biotechnology Information (NCBI) Sequence Read Archive collection PRJNA490626. The reads were mapped to the TM-1 reference genome using the mapping software Hisat2 with default parameters (Pertea et al., 2016). The number of reads for each gene was then counted using HTSeq-Count (Anders et al., 2015), followed by calculation of the Transcripts Per Million (TPM) value of each tissue. The sampling tissues included fibers and ovules at −3, 0, 1, 3, and 5 days, and fibers at 10, 15, 20, and 25 DPA (day post anthesis). When TPM of a gene was >3, it was considered to be expressed during fiber development. We further selected the genes with the higher expression in these tissues rather than other vegetative tissues, and Gene Ontology (GO) analysis was performed online (http://www.omicshare.com/tools) to investigate the function of the candidate genes.

## Results

### Phenotypic Variation for Fiber Quality Traits

**Figure 1:**
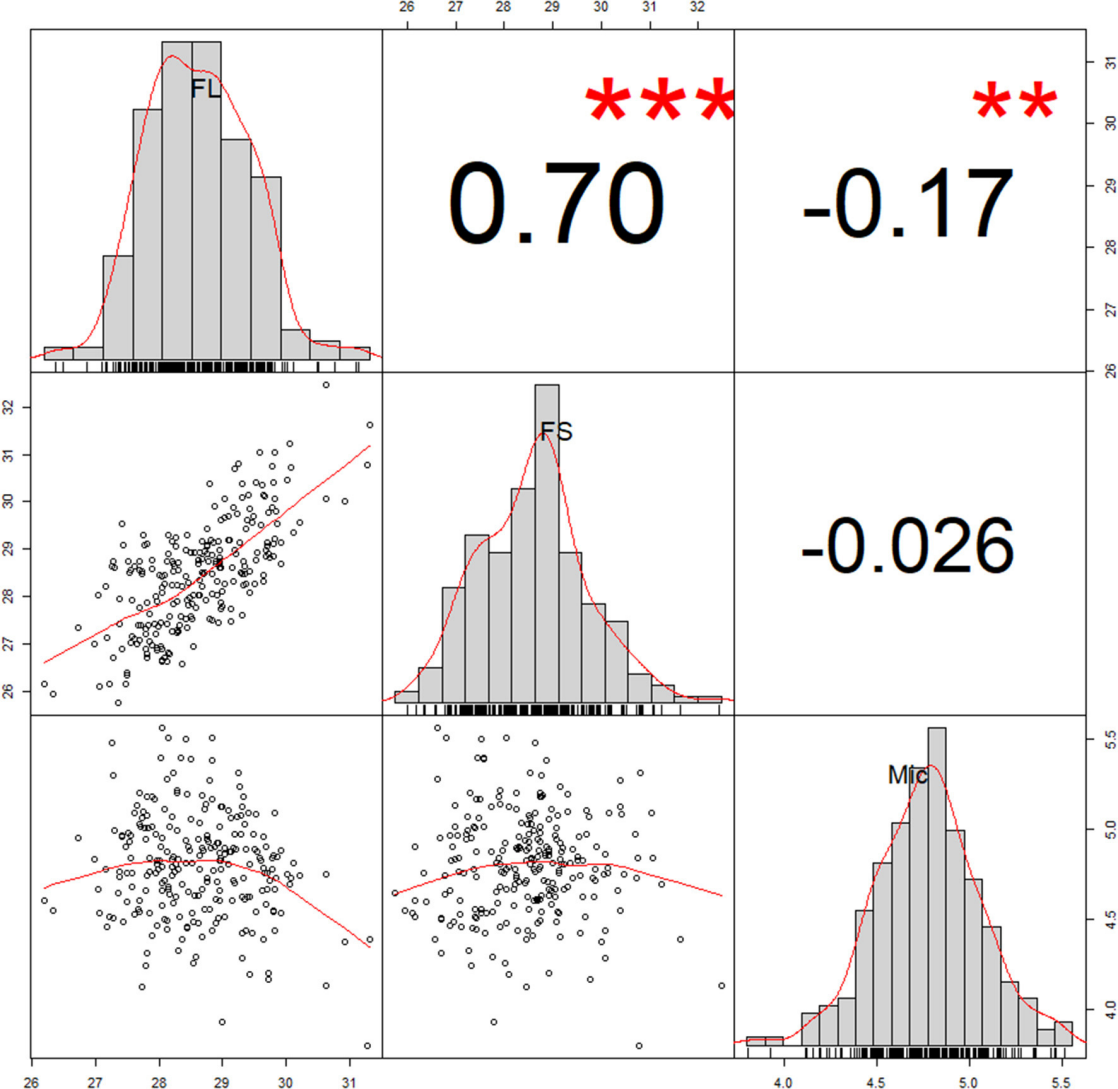
Correlation analysis of three fiber quality traits. The number in these boxes indicated correlation coefficient (R value). The correlation coefficient and the significance between traits were conducted using Best Linear Unbiased Prediction (BLUP) values. **P < 0.01, ***P < 0.001. FL, fiber length; FS, fiber strength; Mic, fiber micronaire.

Fiber quality parameters exhibited significant variation among 242 accessions evaluated in 12 different environments. The phenotypic values of FL, FS, and Mic ranged from 23.41 to 34.30 mm, 21.41 to 41.30 cN·tex^−1^, 2.38 to 6.72, respectively (Table 1). Coefficients of variation (CV) were the largest for Mic (7.09–12.81%), followed by FS (4.90–8.56%), and lowest for FL (3.70–4.80%). The breeding value (marked as BLUP) was applied to eliminate interference of environmental factors on phenotypic value. The BLUP showed that FL, FS, and Mic ranged from 26.19 to 31.31 mm, 25.76 to 32.47 cN·tex^−1^, and 3.80 to 5.56, respectively (Figure 1 and Table 1). The *h*^2^ of FL was 89.55%, FS was 80.95%, and Mic was 84.82%, respectively (Table 1). Correlation analysis revealed that the same trait from different environments had a high correlation (Supplementary Figures 1–3). Based on the BLUP, FL exhibited a high positive correlation with FS and a negative correlation with Mic (Figure 1).

**Table 1:** Phenotypic variation for three fiber quality traits in multiple field environments and their heritability.

Trait^*^	Environment^**^	**Max**	**Min**	**Mean**	**SD**	**CV (%)**	***h*^**2**^ (%)**
FL (mm)	11XJ	32.2	23.4	27.7	1.30	4.69	89.55
	12XJ	32.6	25.6	28.6	1.18	4.13	
	13XJ	31.4	24.9	28.4	1.15	4.07	
	17XJ	34.3	24.0	28.9	1.32	4.56	
	11XX	32.9	25.7	30.1	1.24	4.11	
	12XX	31.1	24.1	27.6	1.19	4.33	
	13XX	31.3	24.6	28.5	1.29	4.51	
	11NY	32.2	25.3	28.6	1.06	3.70	
	12NY	30.5	24.9	27.8	1.03	3.72	
	13NY	31.9	25.6	29.2	1.19	4.06	
	18YC	34.2	26.5	30.7	1.30	4.22	
	18DT	31.8	23.9	27.2	1.31	4.80	
	BLUP	31.3	26.2	28.6	0.86	3.02	
FS (cN·tex^−1^)	11XJ	31.9	22.5	26.3	1.55	5.88	80.95
	12XJ	32.5	23.8	27.0	1.59	5.89	
	13XJ	33.7	21.4	25.5	1.99	7.80	
	17XJ	34.6	22.1	28.4	2.18	7.65	
	11XX	34.7	23.5	29.7	1.57	5.30	
	12XX	35.0	23.8	29.0	1.83	6.32	
	13XX	35.7	25.1	30.3	2.16	7.14	
	11NY	33.4	24.9	28.2	1.38	4.90	
	12NY	32.7	24.2	28.1	1.72	6.14	
	13NY	35.9	24.4	29.5	2.19	7.42	
	18YC	41.3	25.0	31.4	2.69	8.56	
	18DT	35.9	22.5	27.8	2.32	8.33	
	BLUP	32.5	25.8	28.4	1.15	4.03	
Mic	11XJ	6.7	3.5	5.0	0.49	9.76	84.82
	12XJ	5.2	2.6	4.0	0.50	12.54	
	13XJ	5.4	2.7	4.3	0.51	11.85	
	17XJ	6.0	2.4	4.6	0.55	11.97	
	11XX	5.4	2.8	4.3	0.48	11.37	
	12XX	6.0	3.4	5.0	0.42	8.37	
	13XX	5.6	3.4	4.7	0.38	8.11	
	11NY	6.4	3.8	5.1	0.41	7.92	
	12NY	6.2	3.9	5.3	0.38	7.09	
	13NY	5.7	3.3	4.5	0.41	9.04	
	18YC	6.6	4.0	5.4	0.47	8.62	
	18DT	6.4	2.8	5.3	0.68	12.81	
	BLUP	5.6	3.8	4.8	0.29	6.06	

* *FL, fiber length; FS, fiber strength; Mic, fiber micronaire. h^2^, broad-sense heritability.*

** *The 12 environments for the phenotypic investigation include 11, 12, and 13XJ from Korla, Xinjiang in 2011, 2012, and 2013, respectively; 17XJ from Shawan, Xinjiang in 2017; 11, 12, and 13XX from Xinxiang, Henan in 2011, 2012, and 2013, respectively; 11, 12, and 13NY from Nanyang, Henan in 2011, 2012, and 2013, respectively; 18YC from Yancheng, Jiangsu in 2018; 18DT from Dangtu, Anhui in 2018, respectively*.

### Genetic Variation Based on SNPs

We used the CottonSNP80K array to genotype 242 upland cotton accessions and obtained 56,010 high-quality SNPs (Supplementary Table 2), of which 30,933 were located on At subgenome and 25,077 were located on Dt subgenome, with wide distribution on chromosomes (Supplementary Figure 4). A04 and D04 showed the lowest SNP density, while A08 showed the highest SNP density. The polymorphism information content (PIC) values ranged from 0.222 and 0.308 among chromosomes, and the mean PIC of the At and Dt subgenomes were 0.278 and 0.274, respectively (Supplementary Table 2).

### Genome-Wide Association Studies

**Figure 2:**
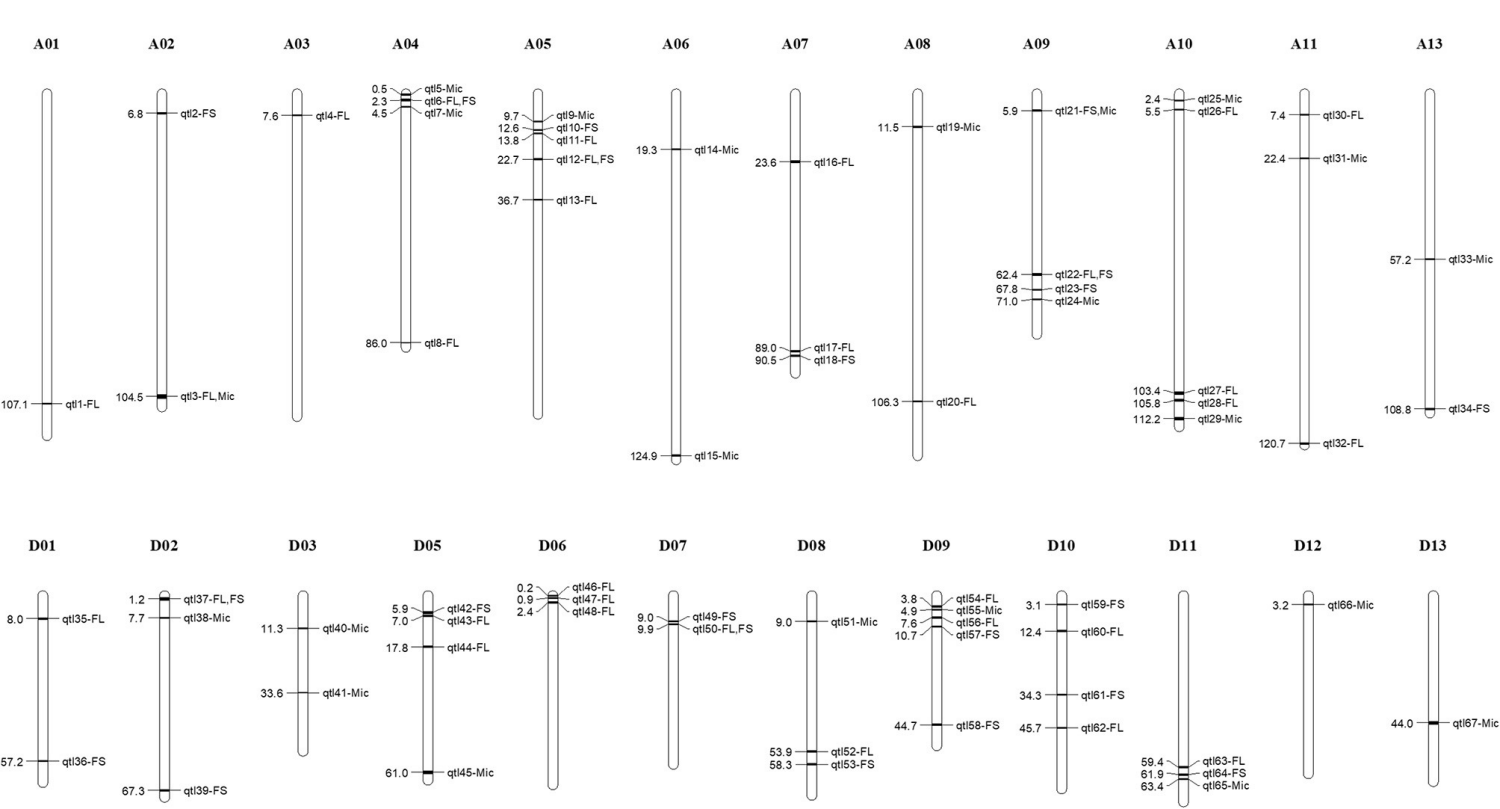
Chromosome distribution of stable quantitative trait loci (QTLs) associated with three fiber quality traits. The numbers on the left of chromosome indicates physical location of the corresponding QTLs. FL, fiber length; FS, fiber strength; Mic, fiber micronaire.

Using multi-locus mixed linear model, a total of 387 QTNs were identified for FL, FS, and Mic. By merging QTN with flanking 200 kb interval as the same QTL, a total of 273 QTLs were obtained (Supplementary Table 3). To reduce false positives, we considered the QTL associated with two or more environments as stable QTL. As a result, 67 stable QTLs were identified, including 31 of FL, 21 of FS, and 22 of Mic, respectively, with 7 QTLs detected simultaneously to be associated with 2 of the 3 traits (Figure 2, Table 2, and Supplementary Table 4). Of them, 34 QTLs co-located with previous studies and 33 QTLs were newly revealed in this study (Table 2). In addition, 17 co-located QTLs were reported to be associated with more than 2 traits in previous studies. For example, qtl15 was related to Mic in this study, but it was also reported to be simultaneously associated with FL, FS, FU, and Mic (Nie et al., 2020). Of seven QTLs detected simultaneously to be associated with two traits, five QTLs were associated simultaneously with FL and FS, one for FL and Mic, and one for FS and Mic, respectively (Supplementary Figure 5). Furthermore, three pairs homologous QTLs, namely, qtl6/qtl45, qtl20/qtl52, and qtl21/qtl55, were detected between A and D subgenomes. These results showed the high genetic stability, significant correlation, and common selection of subgenomes in partial chromosome segments among the three fiber quality traits in the process of breeding.

**Table 2:** Stable QTLs and co-located QTLs identified for fiber length, fiber strength, and fiber micronaire.

**QTL_ID**	**Chromosome**	QTL_start (Mb)^*^	**QTL_end (Mb)**	Associated traits^**^	*R*^**2**^ (%)^***^	Reported associated traits^**^	**References**
qtl1	A01	106.88	107.28	FL	3.32	FS	Ma et al., 2018
qtl2	A02	6.48	7.15	FS	9.39	–	–
qtl3	A02	103.82	105.19	FL	6.31	FS	Li et al., 2018
qtl3	A02	103.82	105.19	Mic	10.12	FS	Li et al., 2018
qtl4	A03	7.44	7.84	FL	15.77	–	–
qtl5	A04	0.21	0.80	Mic	7.55	FS	Huang et al., 2017
qtl6	A04	1.90	2.71	FL	3.12	FL, FU	Nazir et al., 2020
qtl6	A04	1.90	2.71	FS	6.34	FL, FU	Nazir et al., 2020
qtl7	A04	4.28	4.69	Mic	2.66	FS	Fang et al., 2017
qtl8	A04	85.75	86.15	FL	7.76	–	–
qtl9	A05	9.47	9.91	Mic	15.38	FL, FE	Huang et al., 2017; Li et al., 2018; Nazir et al., 2020
qtl10	A05	12.37	12.77	FS	1.71	FS	Li et al., 2018
qtl11	A05	13.54	13.96	FL	3.16	–	–
qtl12	A05	22.44	23.01	FL	4.79	FL	Li et al., 2018
qtl12	A05	22.44	23.01	FS	9.21	FL	Li et al., 2018
qtl13	A05	36.49	36.90	FL	6.43	–	–
qtl14	A06	19.06	19.46	Mic	4.30	–	–
qtl15	A06	124.72	125.16	Mic	10.11	FL, FS, FU, Mic	Nie et al., 2020
qtl16	A07	23.21	24.01	FL	6.48	–	–
qtl17	A07	88.73	89.20	FL	10.82	FL, FS, FE, Mic	Huang et al., 2017; Li et al., 2018; Ma et al., 2018; Dong et al., 2019
qtl18	A07	90.26	90.72	FS	14.63	FL, FS, FE	Sun et al., 2017; Ma et al., 2018; Dong et al., 2019
qtl19	A08	11.26	11.77	Mic	3.19	–	–
qtl20	A08	106.10	106.51	FL	20.15	–	–
qtl21	A09	5.64	6.15	FS	8.31	FE, FS	Ma et al., 2018; Shen et al., 2019
qtl21	A09	5.64	6.15	Mic	3.46	FE, FS	Ma et al., 2018; Shen et al., 2019
qtl22	A09	62.02	62.78	FL	0.63	–	–
qtl22	A09	62.02	62.78	FS	13.88	–	–
qtl23	A09	67.59	68.02	FS	10.28	–	–
qtl24	A09	70.80	71.20	Mic	3.33	–	–
qtl25	A10	2.24	2.64	Mic	12.58	FL	Li et al., 2018
qtl26	A10	5.33	5.73	FL	15.49	FU	Huang et al., 2017
qtl27	A10	102.88	103.92	FL	8.22	FL, FU	Huang et al., 2017
qtl28	A10	105.41	106.23	FL	7.48	FL, FU	Huang et al., 2017
qtl29	A10	111.74	112.65	Mic	7.89	FL, FE, FU	Sun et al., 2017; Ma et al., 2018
qtl30	A11	7.12	7.60	FL	4.46	FE	Li et al., 2018
qtl31	A11	22.23	22.63	Mic	4.09	FL, FE, FU	Ma et al., 2018; Nazir et al., 2020
qtl32	A11	120.50	120.99	FL	4.35	–	–
qtl33	A13	57.03	57.43	Mic	5.31	FS	Ma et al., 2018
qtl34	A13	108.60	109.06	FS	3.79	–	–
qtl35	D01	7.81	8.27	FL	4.12	–	–
qtl36	D01	57.00	57.40	FS	14.70	–	–
qtl37	D02	0.80	1.69	FL	10.28	FS, FE	Huang et al., 2017; Ma et al., 2018
qtl37	D02	0.80	1.69	FS	8.44	FS, FE	Huang et al., 2017; Ma et al., 2018
qtl38	D02	7.49	7.90	Mic	6.79	–	–
qtl39	D02	66.95	67.70	FS	6.87	FL, FU	Liu et al., 2020; Nazir et al., 2020
qtl40	D03	11.10	11.50	Mic	4.58	–	–
qtl41	D03	33.42	33.82	Mic	1.81	–	–
qtl42	D05	5.52	6.32	FS	9.16	FE, Mic	Fang et al., 2017; Ma et al., 2018
qtl43	D05	6.68	7.32	FL	3.96	Mic	Liu et al., 2020
qtl44	D05	17.50	18.09	FL	9.63	FU, FS	Huang et al., 2017; Li et al., 2018
qtl45	D05	60.57	61.47	Mic	6.94	FL, FS, FU	Li et al., 2018
qtl46	D06	0.00	0.42	FL	7.97	FL	Sun et al., 2017
qtl47	D06	0.68	1.09	FL	4.09	FL, FU	Ma et al., 2018; Nazir et al., 2020
qtl48	D06	2.20	2.66	FL	6.04	Mic	Sun et al., 2017
qtl49	D07	8.75	9.15	FS	4.07	–	–
qtl50	D07	9.70	10.11	FL	4.42	–	–
qtl50	D07	9.70	10.11	FS	3.99	–	–
qtl51	D08	8.75	9.15	Mic	1.36	–	–
qtl52	D08	53.61	54.10	FL	5.00	–	–
qtl53	D08	57.96	58.69	FS	3.33	Mic, FS, FE, FU	Ma et al., 2018; Liu et al., 2020; Nazir et al., 2020
qtl54	D09	3.52	4.09	FL	4.43	–	–
qtl55	D09	4.66	5.06	Mic	3.20	–	–
qtl56	D09	7.30	7.85	FL	6.64	–	–
qtl57	D09	10.48	10.95	FS	10.62	–	–
qtl58	D09	44.42	45.01	FS	6.57	–	–
qtl59	D10	2.94	3.34	FS	2.14	FU	Nazir et al., 2020
qtl60	D10	12.02	12.69	FL	5.49	–	–
qtl61	D10	34.10	34.50	FS	6.02	–	–
qtl62	D10	45.47	45.87	FL	3.24	FE	Ma et al., 2018
qtl63	D11	59.13	59.65	FL	5.33	–	–
qtl64	D11	61.65	62.09	FS	15.61	FU	Nazir et al., 2020
qtl65	D11	63.20	63.60	Mic	13.73	–	–
qtl66	D12	2.97	3.40	Mic	17.57	FU	Li et al., 2018
qtl67	D13	43.58	44.46	Mic	14.00	–	–

* *The physical location of the quantitative trait loci (QTL) is from the TM-1 V2.1 genome as reference (Hu et al., 2019).*

** *FL, fiber length; FS, fiber strength; Mic, fiber micronaire; FU, fiber uniformity; FE, fiber elongation.*

*** *R^2^ (%) indicates the percentage of phenotypic variation explained by each QTL*.

### Identification of Candidate Genes Within QTL

**Figure 3:**
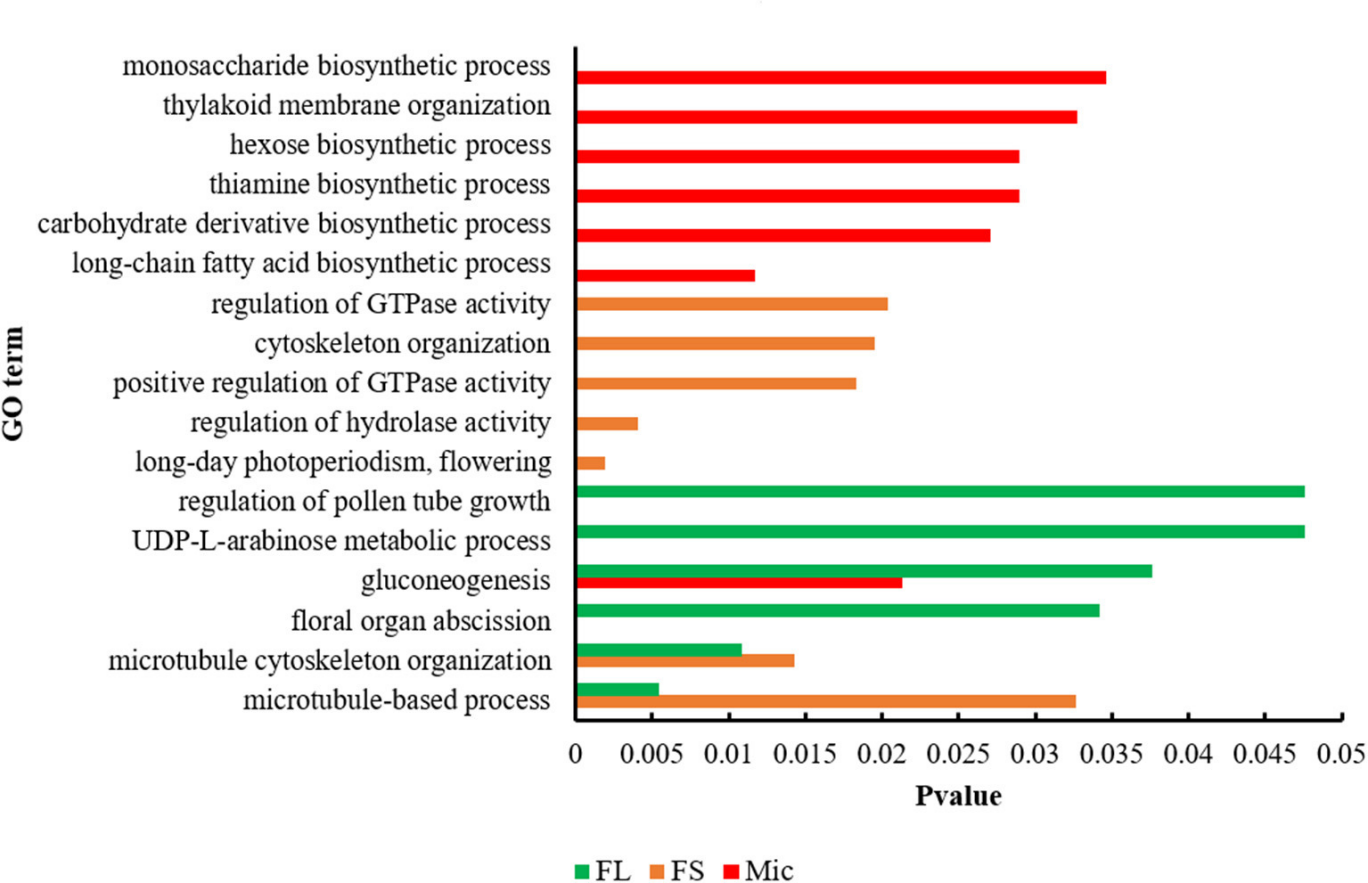
Function characterization of candidate genes associated with three fiber quality traits by Gene Ontology (GO) analysis. FL, fiber length; FS, fiber strength; Mic, fiber micronaire.

The candidate genes in the QTL intervals were extracted, and their expression patterns during the fiber development were analyzed based on the released *G. hirsutum* TM-1 genome (Hu et al., 2019). As a result, 649 candidate genes were identified, with 256 of FL, 244 of FS, and 149 of Mic (Supplementary Table 5). GO analysis was then conducted to explore the biological functions of the candidate genes (Figure 3 and Supplementary Table 6). For FL, the enriched processes contained regulation of pollen tube growth, gluconeogenesis, and UDP-L-arabinose metabolic process. For FS, the candidate genes were involved in kinase activity and flowering related process, including regulation of hydrolase activity, regulation of GTPase activity, and long-day photoperiodism. GO terms related to microtubule and cytoskeleton organization were also enriched in FL and FS, respectively. For Mic, the genes were mainly enriched in glycol-metabolism related processes, such as gluconeogenesis, carbohydrate derivative biosynthetic process, hexose biosynthetic process, and monosaccharide biosynthetic process. Besides, other processes closely related to fiber development were also enriched, such as the long-chain fatty acid biosynthetic process, thiamine biosynthetic process, and thylakoid membrane organization.

### Key QTLs/Genes Associated With Fiber Quality

Of the stable QTLs, a QTL hotspot (D06: 0–1.09 Mb) which contained two QTLs, namely, qtl46 and qtl47, both associated with FL, were found (Figure 2 and Table 2). This QTL hotspot had also been reported to be related to FL in previous studies (Sun et al., 2017; Ma et al., 2018). In the QTL hotspot region, 24 candidate genes were identified, and most of them were involved in fiber development, such as *GH_D06G0024* encoded gibberellin-regulated family protein and *GH_D06G0025* encoded SNARE-like superfamily protein (Supplementary Table 5). We further focused on QTLs associated with multiple traits (Figure 2, Supplementary Figure 5, and Table 2). Three genes (*GH_A05G2302, GH_A05G2316*, and *GH_A08G0793*) that expressed highly from 10 to 25 DPA during fiber development were found in QTLs associated with both FL and FS (Supplementary Table 5). The *GH_A05G2302* encoded NAD(P)-binding Rossmann-fold superfamily protein involved in sphingolipid biosynthetic process (Chao et al., 2011). The *GH_A05G2316* encoded Glycosyl hydrolase superfamily protein, and was related to the carbohydrate metabolic process (Nibbering et al., 2020). The *GH_A08G0793* encoded C2H2-type zinc finger family protein, which also plays a crucial role in fiber development (Salih et al., 2019).

**Figure 4:**
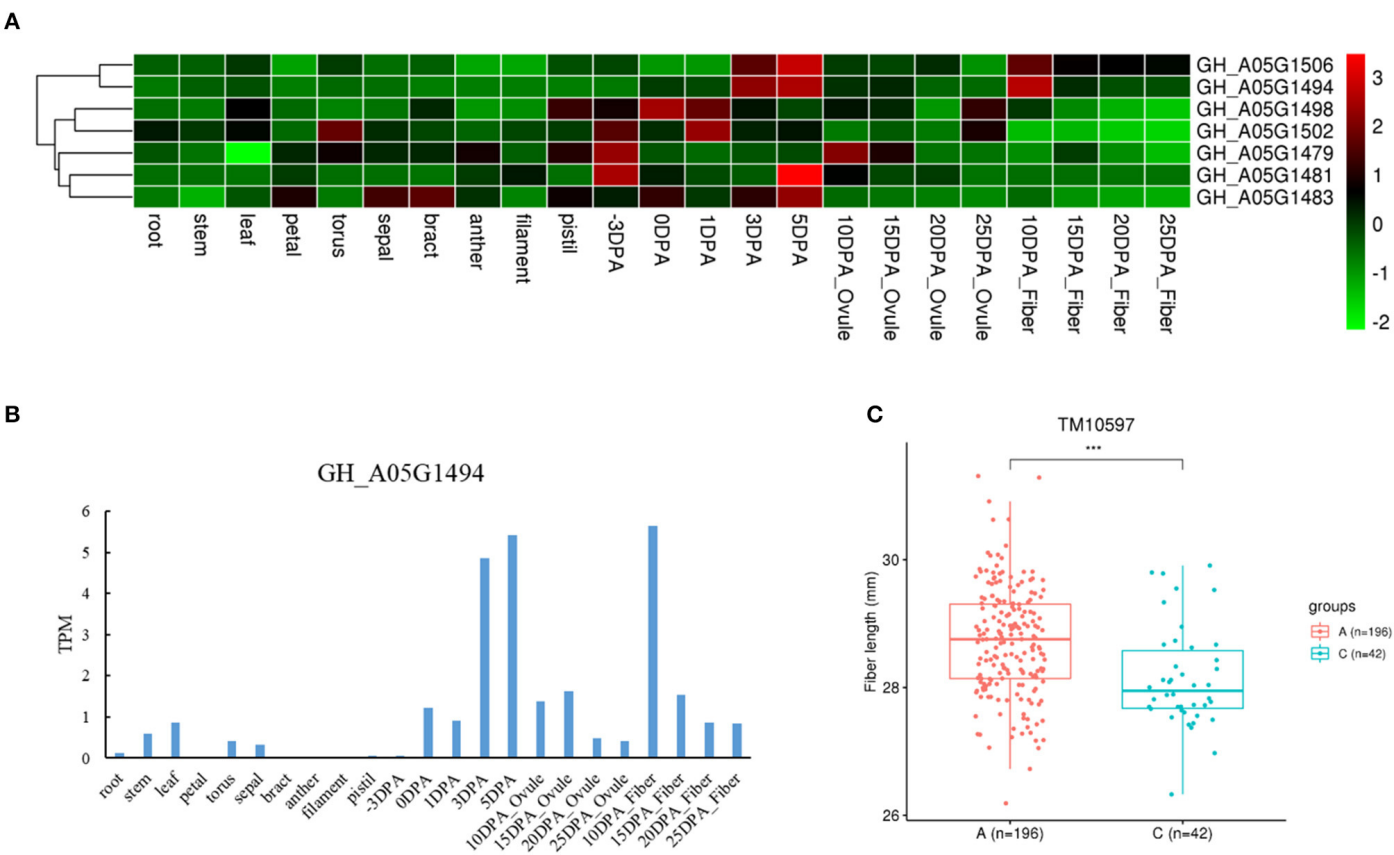
Quantitative trait loci (QTLs) and candidate genes located on qtl11 on chromosome A05. **(A)** Expression heatmap of candidate genes in the qtl11. **(B)** The expression patterns of the *GH_A05G1494* in different tissues. **(C)** Boxplots for the phenotypic values of quantitative trait nucleotide (QTN) in UTR region of the *GH_A05G1494*. ***P < 0.001.

**Figure 5:**
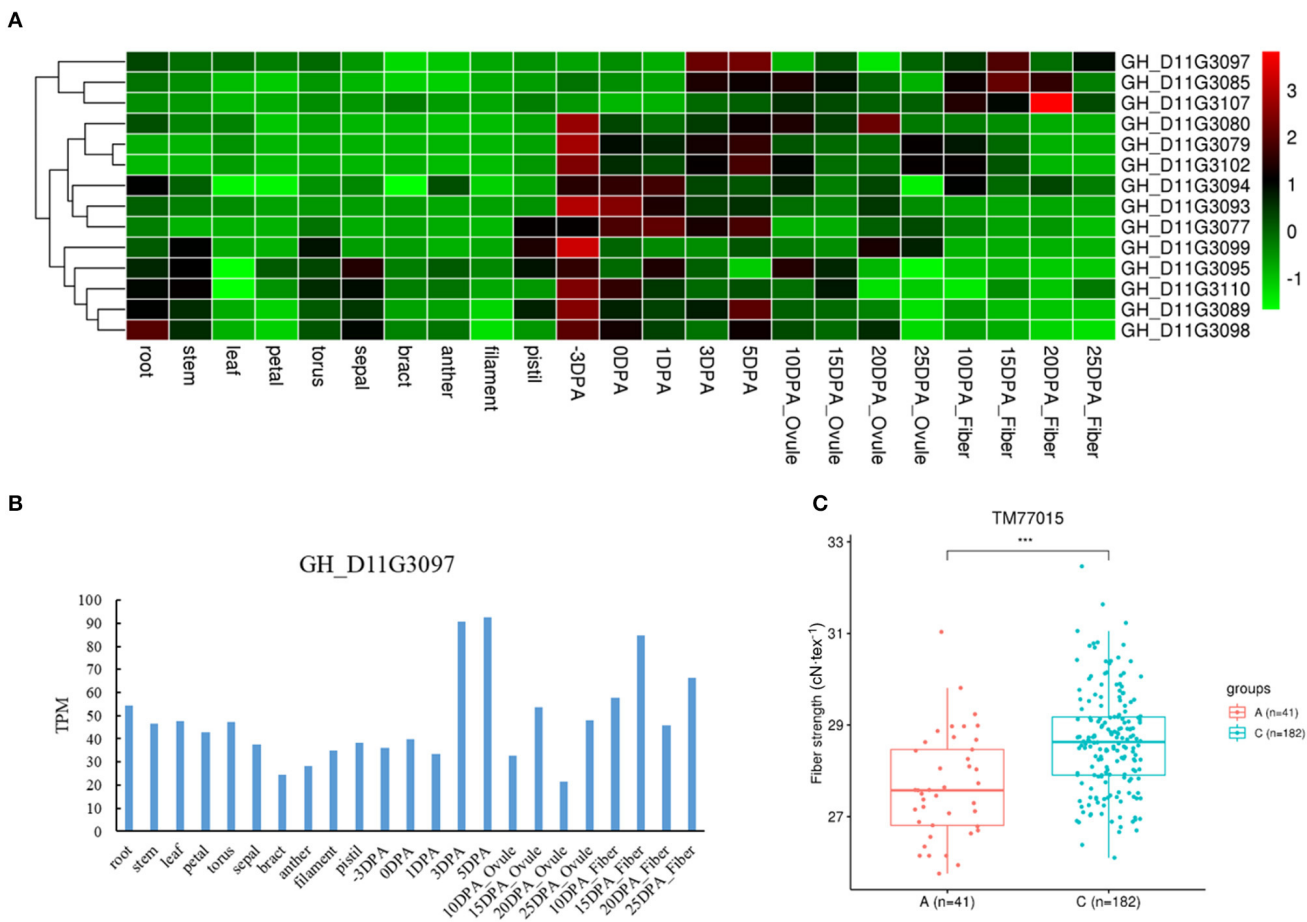
Quantitative trait loci (QTLs) and candidate genes located on qtl64 on chromosome D11. **(A)** Expression heatmap of candidate genes in the qtl64. **(B)** The expression patterns of the *GH_D11G3097* in different tissues. **(C)** Boxplots for the phenotypic values of quantitative trait nucleotide (QTN) in exon of the *GH_D11G3097*. ***P < 0.001.

**Figure 6:**
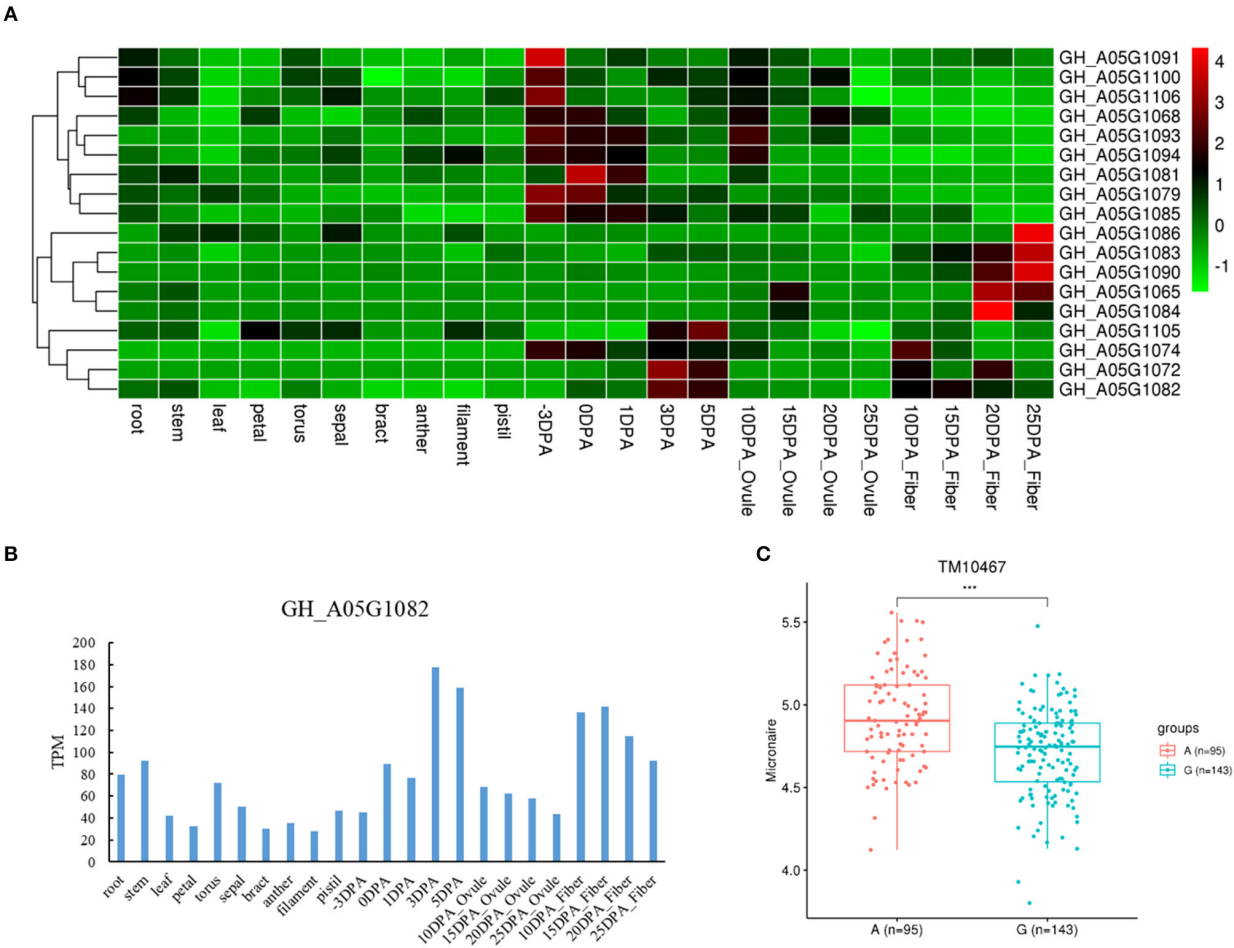
Quantitative trait loci (QTLs) and candidate genes located on qtl9 on chromosome A05. **(A)** Expression heatmap of candidate genes in the qtl9. **(B)** The expression patterns of the *GH_A05G1082* in different tissues. **(C)** Boxplots for the phenotypic values of quantitative trait nucleotide (QTN) in exon of the *GH_A05G1082*. ***P < 0.001.

We also found three genes with different haplotypes to be significantly related to the corresponding fiber quality trait. The qtl11 (A05: 13.54–13.96 Mb) on chromosome A05 was associated with FL, and seven candidate genes were detected in the interval (Figure 4A and Table 2). Of the genes, the *GH_A05G1494* encoded an SGS domain-containing protein with high expression at 3DPA, 5 DPA, and 10DPA during fiber development (Figure 4B), which was related to tubulin-binding and ubiquitin-protein ligase binding. A QTN (TM10597) in UTR of *GH_A05G1494* was then used to investigate the correlation between the two haplotypes and FL phenotype. The FL values with an A genotype were significantly higher than that with a C genotype (Figure 4C), implying its vital role in improving FL. The qtl64 (D11: 61.65–62.09 Mb) on chromosome D11 was associated with FS and 14 candidate genes were detected (Figure 5A, Supplementary Figure 6, and Table 2). The *GH_D11G3097* (named as *PBG1*) encoded 20S proteasome beta subunit G1, and was related to the ubiquitin-dependent protein catabolic process. *Via* tissue and organ transcriptome profiling, PBG1 was highly expressed at 3 DPA, 5 DPA, 15 DPA, and 25 DPA of fiber development (Figure 5B). We also found that a QTN (TM77015) in *GH_D11G3097* exon produced two different haplotypes. The FS values with a C genotype were significantly higher than that with an A genotype (Figure 5C), indicating its potential functions in increasing FS. For Mic, the *GH_A05G1082* (named as *RANBP1*) from qtl9 (A05: 9.47–9.91 Mb) on chromosome A05 was identified (Figure 6A, Supplementary Figure 7, and Table 2). RANBP1 encoded RAN binding protein 1 with a molecular function of GTPase activator activity, which was highly expressed at 3 DPA to 5 DPA and 10 DPA to 25 DPA of fiber development, respectively (Figure 6B). By analyzing the QTN TM10467 located in the gene region, the Mic value with a G genotype was found to be significantly finer than that with an A genotype (Figure 6C).

## Discussion

The need for elite cultivars with higher fiber quality has further increased with machine-picking of cotton and textile technology improvement. FL, FS, and Mic are critical fiber traits to evaluate cotton fiber quality. Thus, QTLs and candidate genes related to fiber quality traits may accelerate cotton genetic improvement. With the development of high-density cotton array (Hulse-Kemp et al., 2015; Cai et al., 2017) and genomic re-sequencing analysis, numerous QTLs and genes associated with fiber quality traits were identified (Sun et al., 2017; Ma et al., 2018; Su et al., 2020). However, due to the complex nature of quantitative traits, the stability and validity these QTLs still need to be further verified across multiple environments. Exploring the stable loci and candidate genes involved in fiber quality traits by integrating multiple field environments phenotype data will promote fiber quality, breeding accuracy, and efficiency.

In the current study, we observed large phenotypic variations for FL, FS, and Mic in natural populations of 242 genotypes for multiple years. Compared to yield and components (Su et al., 2016; Nie et al., 2020), fiber quality traits had a higher heritability of more than 80%, consistent with previous reports (Zhang et al., 2019; Liu et al., 2020). Correlation analysis showed that each trait had high correlations in different environments, indicating the genetic stability of fiber quality traits. Furthermore, FL was correlated positively with FS and correlated negatively with Mic, which was also consistent with previous studies (Sun et al., 2017; Ma et al., 2018). These results indicate that fiber quality traits are more suitable for genetic improvement by molecular breeding.

By combining phenotype data from multiple environments with genotypic data, stable QTLs and candidate genes for fiber quality may be revealed through GWAS analysis. In contrast to traditional QTL mapping, GWAS analysis can simultaneously locate multiple traits with greater precision. GWAS has been widely applied in other crops, such as wheat (Juliana et al., 2019), rice (Chen et al., 2014), soybean (Lu et al., 2020), and maize (Wang et al., 2020). In cotton, a large number of associated loci for fiber quality have also been reported *via* GWAS analysis (Zhang et al., 2019; Liu et al., 2020). Nevertheless, multi-year and -environment phenotype investigation is more effective to mine stable QTLs and genes related to fiber quality due to the complex genetic nature of quantitative traits. In the current study, 67 stable QTLs were identified with 33 novel and 34 co-located with previously reported QTLs. The function analysis of 649 candidate genes identified in the stable QTL intervals indicated that numerous enriched processes were closely related to fiber development, including sugar biosynthesis, sugar metabolism, microtubule, and cytoskeleton organization (Whittaker and Triplett, 1999; Li et al., 2002; Zhang et al., 2015). Several co-located QTLs, such as qtl9, qtl17, and qtl18, were also found in previous reports, indicating stable and effective contribution to fiber development. In qtl9 region, the *GH_A05G1083* encoded a Kinase interacting (KIP1-like) family protein. It has been reported that KIP-related protein could regulate cell elongation by affecting the expression of genes involved in plant cell wall organization (Jégu et al., 2013; Li et al., 2020). Furthermore, the *GH_A07G2172* from qtl17, encoded a heat shock protein 89 (HSP89). In cotton, HSP inhibition can disturb the H_2_O_2_ balance and lead to the generation of oxidative stress, which suppresses cotton fiber development (Sable et al., 2018). The qtl20 which explained high phenotypic variation (20.15%) for FL was also identified as a novel QTL in this study. Interestingly, only one gene with the unknown function was found in qtl20 interval. The candidate gene and the related mechanism in this QTL interval is worth further studying for fiber quality improvement.

Identifying key genes involved in fiber quality will accelerate the process of fiber quality breeding in cotton. The *GH_A05G1494* located in qtl11 encoded an SGS domain-containing protein, and highly expressed at 3 DPA, 5 DPA, and 10 DPA during fiber development. Function analysis showed that *GH_A05G1494* was involved in tubulin-binding and ubiquitin-protein ligase binding. The tubulin plays a vital role in cotton cell elongation and cellulose biosynthesis (Whittaker and Triplett, 1999; Li et al., 2002), and protein ubiquitination also plays key roles in plant developmental and plant-environment interactions (Shu and Yang, 2017). Furthermore, the *GH_A05G1082* identified in qtl9 encoded RAN binding protein 1 with GTPase activator activity (Lee et al., 2007), and was also predominantly expressed from 3 DPA to 25 DPA during fiber development, consistent with the previous study (Trainin et al., 1996; Kim and Triplett, 2004). GTPase is widely reported as an essential regulator of cotton fiber elongation, such as GhRac1 (Kim and Triplett, 2004) and GhGRAM (Ye et al., 2021). In addition, two candidate genes were identified in a QTL hotspot (qtl46 and qlt47). The two genes were also highly expressed during fiber development. The *GH_D06G0024* encoded gibberellin-regulated family protein, and gibberellin had an important role in cotton fiber development (Ma et al., 2018). The *GH_D06G0025* was related to vesicle-mediated transport, and vesicles were involved in the transport of growth substances in fiber and promoted the elongation of fiber cells (Yu et al., 2019). The candidate genes identified in the current study might contribute to cotton fiber quality improvement through speed breeding with accuracy and efficiency.

## Conclusions

In this study, a set of natural populations comprising 242 upland cotton accessions showed large phenotypic variation for FL, FS, and Mic value. The *h*^2^ of the three traits further indicated the potential of genetic improvement for cotton fiber quality through molecular breeding. Combined with multi-year and -environment phenotypic data, we identified 67 stable QTLs related to the 3 fiber quality traits, with 33 novel and 34 co-located with previous reported QTLs. The function analysis of candidate genes in the stable QTL regions exhibited that numerous enriched processes were closely related to fiber development, including sugar biosynthesis, sugar metabolism, microtubule, and cytoskeleton organization. Integrated with transcriptome analysis, several fiber predominantly expressed genes were indicated to have potential for fiber quality improvement in cotton, such as *GH_A05G1494* and *GH_A05G1082*.

## Data Availability Statement

The datasets presented in this study can be found in online repositories. The names of the repository/repositories and accession number(s) can be found in the article/Supplementary Material.

## Author Contributions

Experiments were designed by WG. Experiments were performed by XS, GZ, SH, YR, and WL. XS and GZ drafted the manuscript, and WG and MA revised the manuscript. All authors read and approved the final manuscript.

## Conflict of Interest

The authors declare that the research was conducted in the absence of any commercial or financial relationships that could be construed as a potential conflict of interest.

## Publisher's Note

All claims expressed in this article are solely those of the authors and do not necessarily represent those of their affiliated organizations, or those of the publisher, the editors and the reviewers. Any product that may be evaluated in this article, or claim that may be made by its manufacturer, is not guaranteed or endorsed by the publisher.

## Supplementary Material

Supplementary Table 1 Information of 242 upland cotton accessions used in this study.

Supplementary Table 2 Information of high-quality SNPs genotyped by CottonSNP80K array.

Supplementary Table 3 QTNs and QTLs of fiber length (FL), fiber strength (FS), and fiber micronaire (Mic) detected by multi-loci MLM model.

Supplementary Table 4 The information of QTL and QTNs in the QTL interval.

Supplementary Table 5 Expression profile of candidate genes related to fiber length (FL), fiber strength (FS), and fiber micronaire (Mic).

Supplementary Table 6 GO analysis of genes in candidate QTL intervals related to fiber length (FL), fiber strength (FS), and fiber micronaire (Mic).

Supplementary Figure 1 Correlation analysis of fiber length from different environments. The number in these boxes indicated correlation coefficient (R value). ^***^indicated *P* value at the 0.001 levels.

Supplementary Figure 2 Correlation analysis of fiber strength from different environments. The number in these boxes indicated correlation coefficient (R value). ^*^indicated *P* value at the.05 levels, ^**^indicated *P* value at the 0.01 levels, ^***^indicated *P* value at the 0.001 levels.

Supplementary Figure 3 Correlation analysis of fiber micronaire from different environments. The number in these boxes indicated correlation coefficient (R value). ^*^indicated *P* value at the 0.05 levels, ^**^indicated *P* value at the 0.01 levels, ^***^indicated *P* value at the 0.001 levels.

Supplementary Figure 4 Density distribution map of 56010 high-quality SNPs in 26 chromosomes.

Supplementary Figure 5 Venn diagram of QTLs associated with fiber length (FL), fiber strength (FS), and fiber micronaire (Mic).

Supplementary Figure 6 Manhattan plot of FL on Chr. D11. Black dashed line represents the significance threshold (LOD score = 3). Pink dot indicates the position of TM77015.

Supplementary Figure 7 Manhattan plot of Mic on Chr. A05. Black dashed line represents the significance threshold (LOD score = 3). Pink dot indicates the position of TM10467.

## Abbreviations

BLUP: The best linear unbiased prediction
FL: Fiber length
FS: Fiber strength
Mic: Fiber micronaire
GO: Gene ontology
GWAS: Genome-wide association studies
MAF: Minor allele frequency
QTLs: Quantitative trait loci
QTNs: quantitative trait nucleotides
SNP: Single nucleotide polymorphism.
