# Supplementary figures and images for: Genome-Wide Association Analysis Reveals Loci and Candidate Genes Involved in Fiber Quality Traits Under Multiple Field Environments in Cotton (Gossypium hirsutum)
Source: Front Plant Sci. 2021 Aug 5;12:695503. doi: 10.3389/fpls.2021.695503 (PMC8374309; doi:10.3389/fpls.2021.695503)

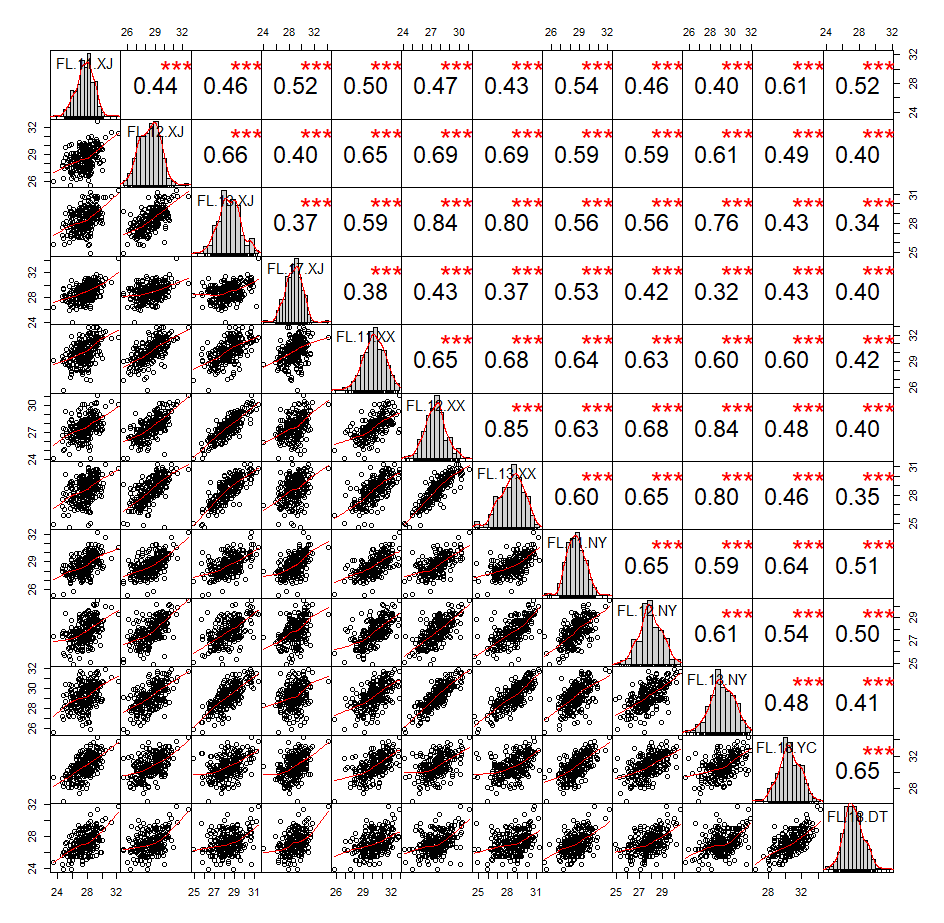

Supplement: Supplementary Figure 1 — Correlation analysis of fiber length from different environments. The number in these boxes indicated correlation coefficient (R value). ***indicated P value at the 0.001 levels. [file Image_1.TIFF]

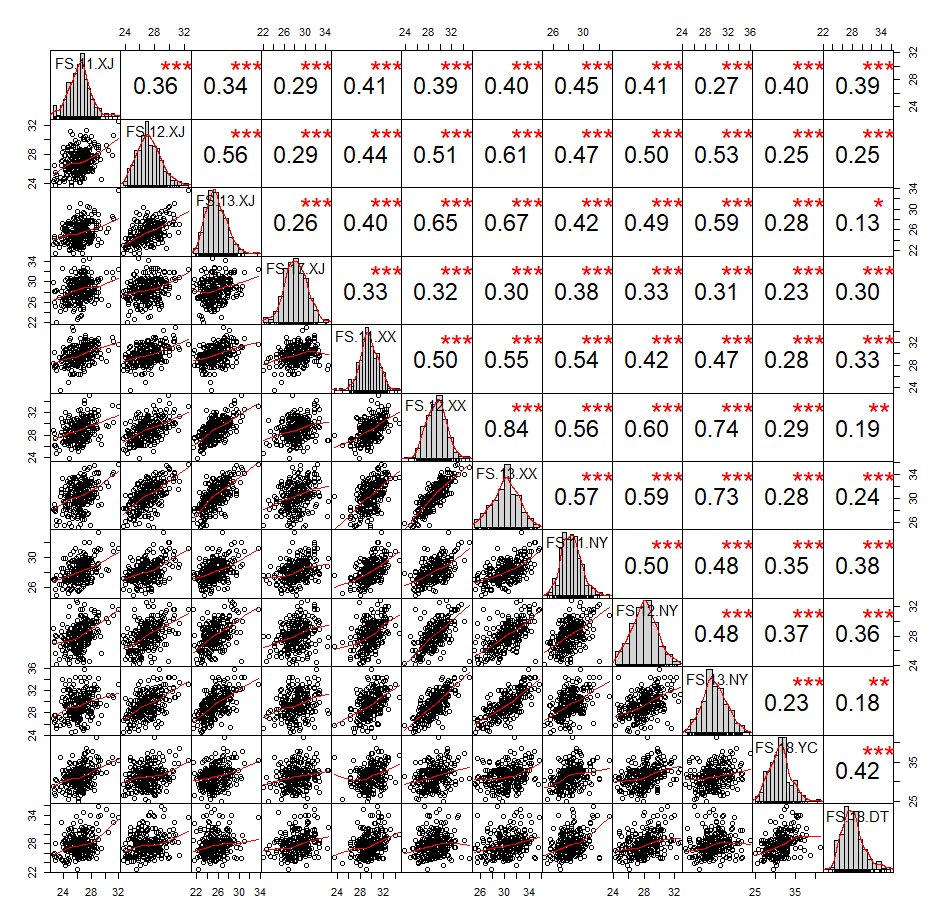

Supplement: Supplementary Figure 2 — Correlation analysis of fiber strength from different environments. The number in these boxes indicated correlation coefficient (R value). *indicated P value at the.05 levels, **indicated P value at the 0.01 levels, ***indicated P value at the 0.001 levels. [file Image_2.TIFF]

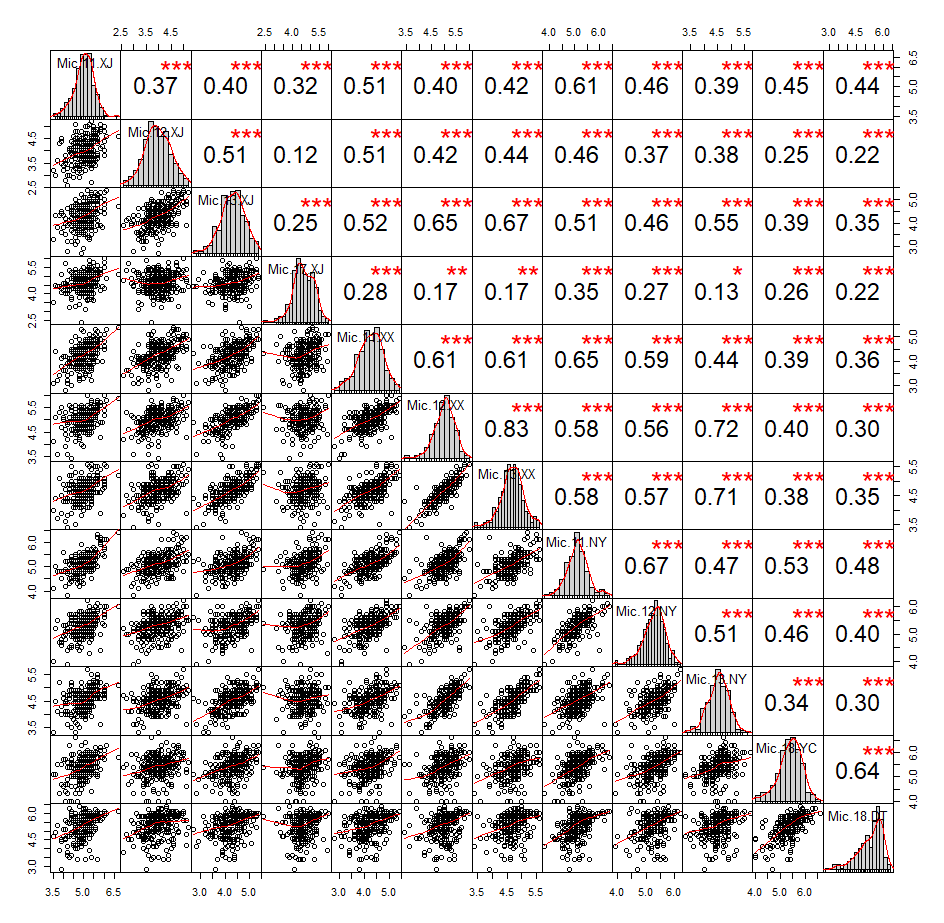

Supplement: Supplementary Figure 3 — Correlation analysis of fiber micronaire from different environments. The number in these boxes indicated correlation coefficient (R value). *indicated P value at the 0.05 levels, **indicated P value at the 0.01 levels, ***indicated P value at the 0.001 levels. [file Image_3.TIFF]

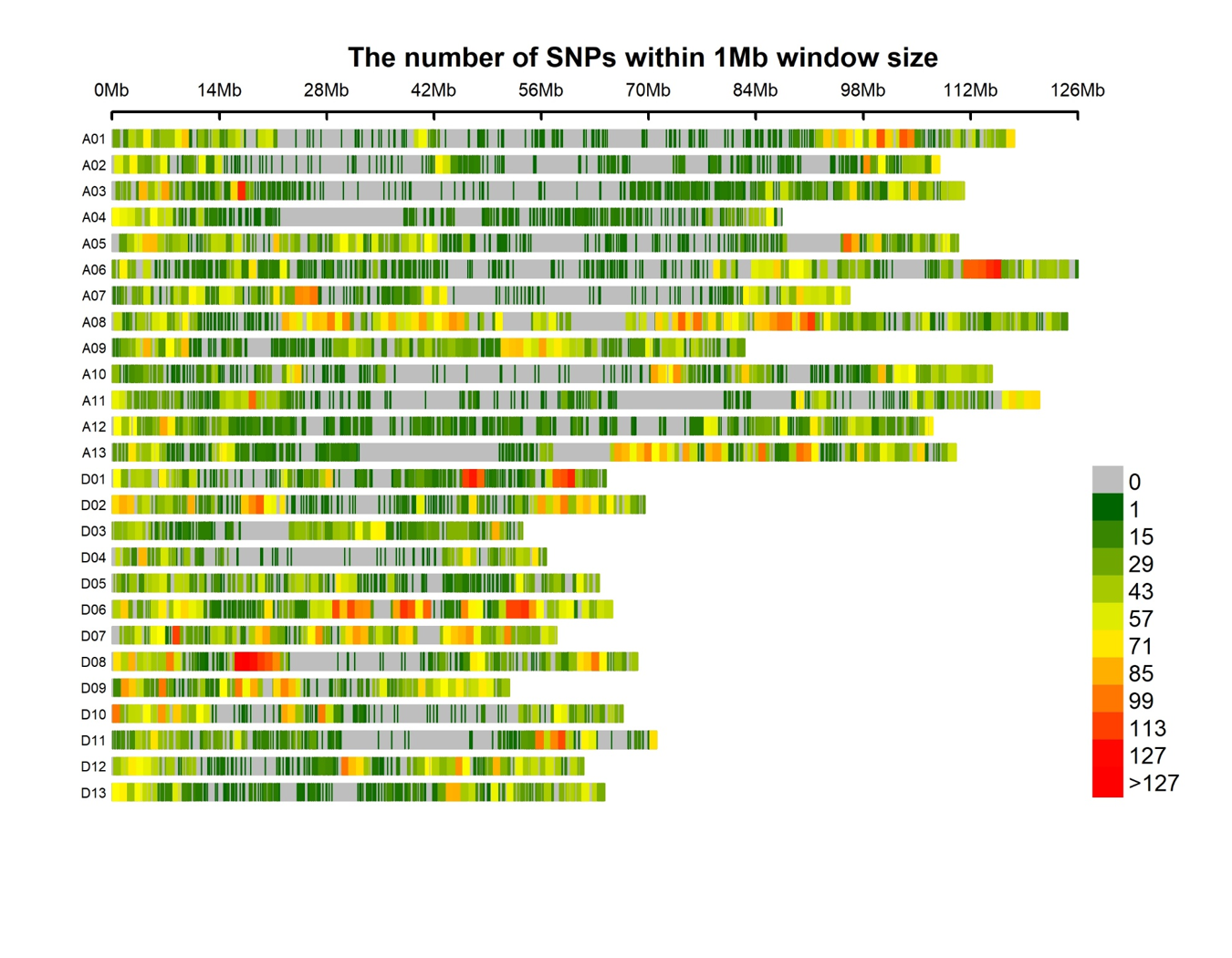

Supplement: Supplementary Figure 4 — Density distribution map of 56010 high-quality SNPs in 26 chromosomes. [file Image_4.TIFF]

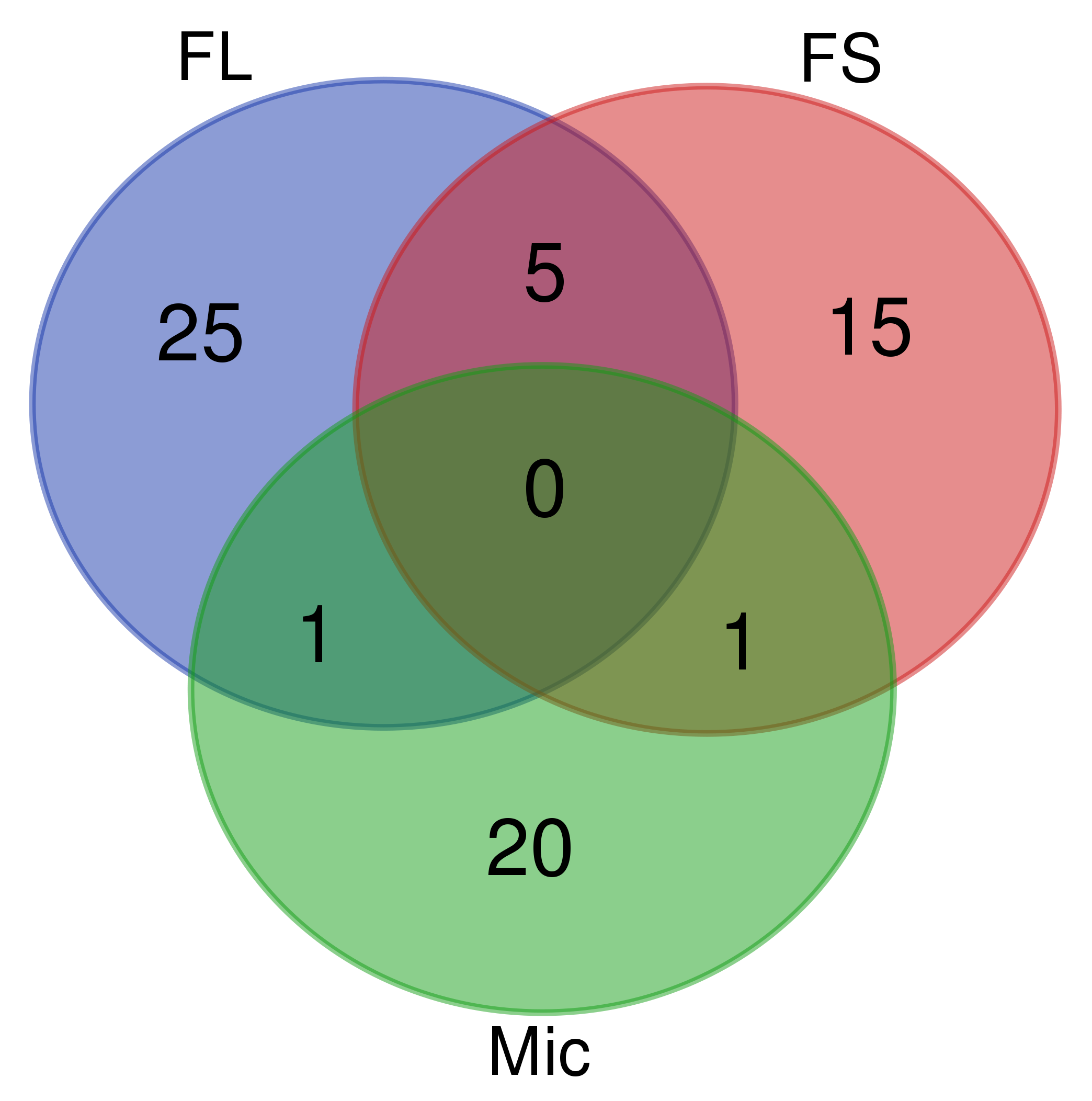

Supplement: Supplementary Figure 5 — Venn diagram of QTLs associated with fiber length (FL), fiber strength (FS), and fiber micronaire (Mic). [file Image_5.TIFF]

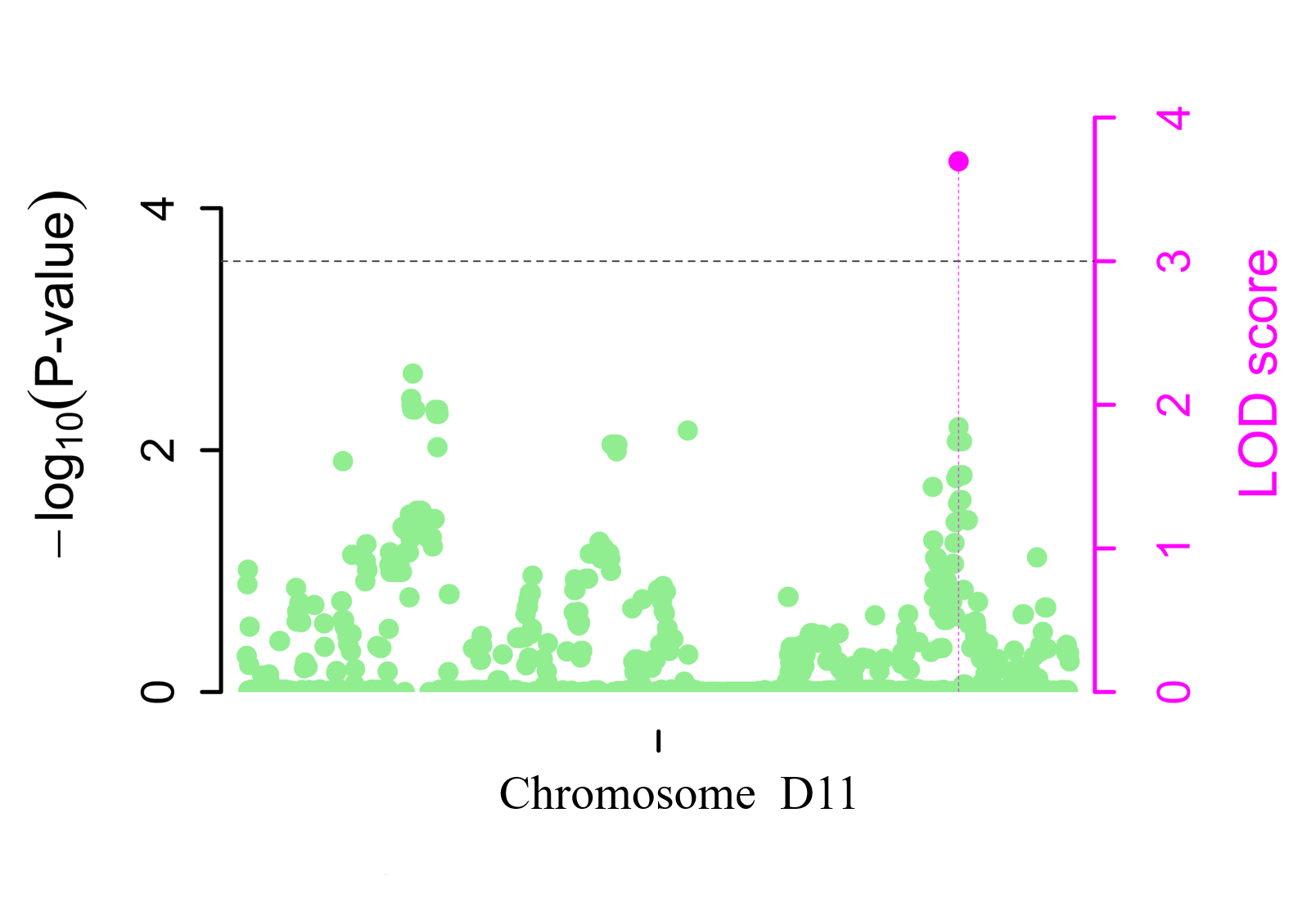

Supplement: Supplementary Figure 6 — Manhattan plot of FL on Chr. D11. Black dashed line represents the significance threshold (LOD score = 3). Pink dot indicates the position of TM77015. [file Image_6.TIFF]

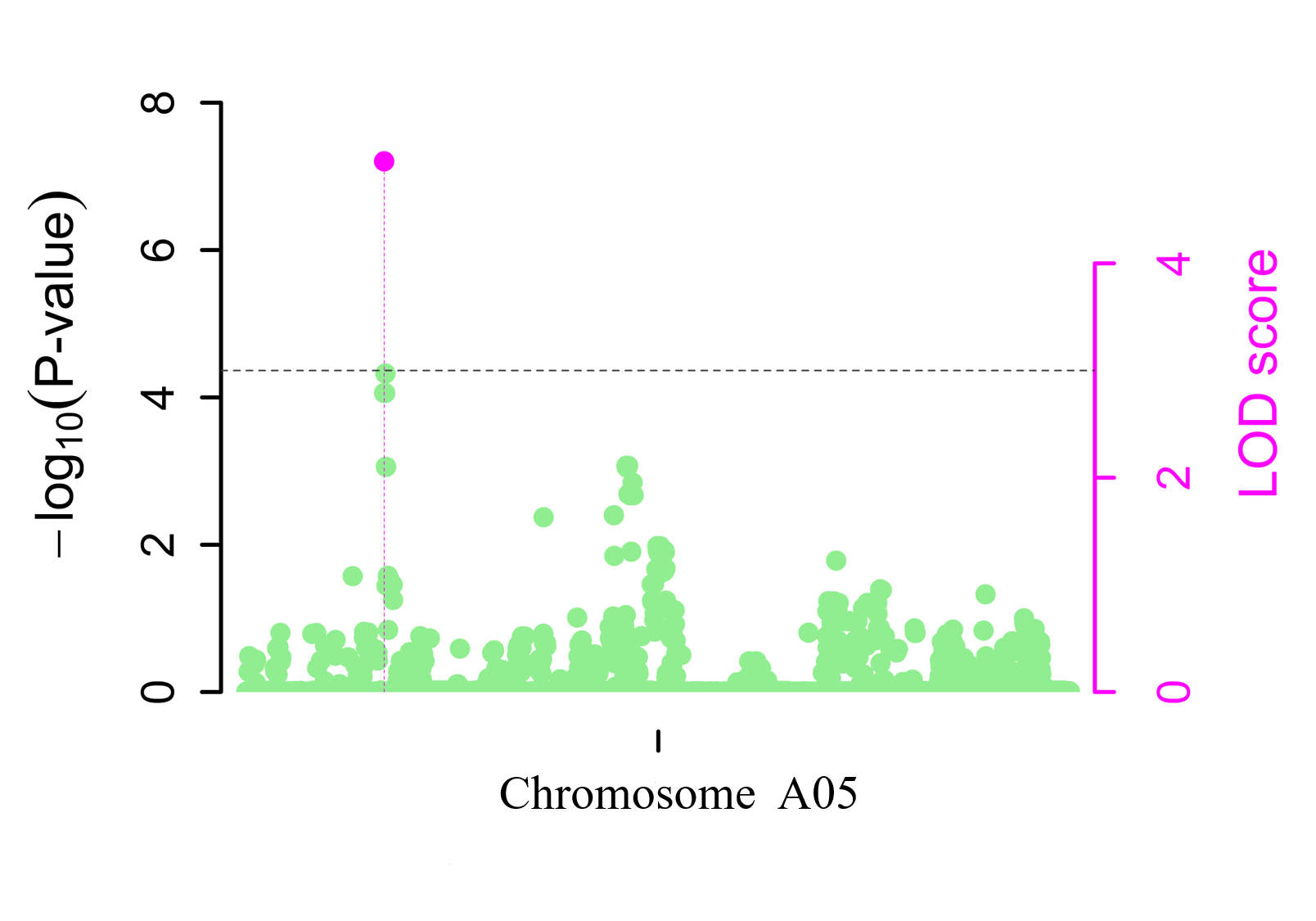

Supplement: Supplementary Figure 7 — Manhattan plot of Mic on Chr. A05. Black dashed line represents the significance threshold (LOD score = 3). Pink dot indicates the position of TM10467. [file Image_7.TIFF]
